# Predictors of incident reversible and potentially reversible cognitive frailty among Taiwanese older adults

**DOI:** 10.1186/s12877-023-03741-4

**Published:** 2023-01-13

**Authors:** Hei-Fen Hwang, Lalu Suprawesta, Sy-Jou Chen, Wen-Yu Yu, Mau-Roung Lin

**Affiliations:** 1 Department of Nursing, National Taipei University of Nursing and Health Sciences, Taipei, Taiwan, ROC; 2 Institute of Injury Prevention and Control, College of Public Health, Taipei Medical University, 250 Wu-Hsing Street, Taipei, 11031 Taiwan, ROC; 3 Department of Sport and Health Education, Faculty of Sport Science and Public Health, Universitas Pendidikan Mandalika, Mataram, West Nusa Tenggara Indonesia; 4 Department of Emergency Medicine, Tri-Service General Hospital, National Defense Medical Center, Taipei, Taiwan, ROC; 5 Department of Emergency Medicine, Taipei Medical University Hospital, Taipei, Taiwan, ROC

**Keywords:** Cognitive frailty, Risk factors, Gait variability, Gait velocity, Older adults

## Abstract

**Background:**

Few studies emphasize on predictors of incident cognitive frailty (CF) and examine relationships between various gait characteristics and CF. Therefore, we conducted a 2-year prospective study to investigate potential predictors, including gait characteristics, of incident reversible CF (RCF) and potentially RCF (PRCF) among Taiwanese older adults.

**Methods:**

Eligible participants were individuals aged ≥ 65 years, who could ambulate independently, and did not have RCF/PRCF at the baseline. The baseline assessment collected information on physical frailty and cognitive measures, in addition to sociodemographic and lifestyle characteristics, preexisting comorbidities and medications, gait characteristics, Tinetti’s balance, balance confidence as assessed by Activities-specific Balance Confidence (ABC) scale, and the depressive status as assessed by the Geriatric Depression Scale. The Mini-Mental State Examination (MMSE), Mattis Dementia Rating Scale, and Digit Symbol Substitution Test were used to evaluate cognitive functions. Incident RCF and PRCF were ascertained at a 2-year follow-up assessment.

**Results:**

Results of the multinomial logistic regression analysis showed that incident RCF was significantly associated with older age (odds ratio [OR] = 1.05) and lower ABC scores (OR = 0.97). Furthermore, incident PRCF was significantly associated with older age (OR = 1.07), lower ABC scores (OR = 0.96), the presence of depression (OR = 3.61), lower MMSE scores (OR = 0.83), slower gait velocity (OR = 0.97), and greater double-support time variability (OR = 1.09).

**Conclusions:**

Incident RCF was independently associated with older age and lower balance confidence while incident PRCF independently associated with older age, reduced global cognition, the presence of depression, slower gait velocity, and greater double-support time variability. Balance confidence was the only modifiable factor associated with both incident RCF and PRCF.

## Introduction

Physical frailty and cognitive impairment often coexist in older adults [1]. Frail older persons with cognitive impairment display higher progression rates of all types of dementia [2] and increased risks of functional disabilities, a poor quality of life, and mortality [3] than those with normal cognitive functioning. Bidirectional associations of physical frailty and cognitive impairment result in a combination of the two geriatric conditions [4]. Considering cognitive and physical dimensions as separate entities may hinder the understanding of common underlying mechanisms and the potential for integrated strategies for prevention and treatment.

Cognitive frailty (CF) was originally defined as having the simultaneous presence of physical frailty and mild cognitive impairment (MCI) in the absence of dementia or preexisting brain disorders [5]. This definition presumes that individuals with CF have additionally greater risk of incident neurocognitive disorders in comparison with those with MCI or physical frailty alone [6]. The prevalence of CF based on this definition is between 1 and 5% in community-dwelling older people; however, the low prevalence suggests limited clinical utility of this concept [7]. A new definition broadened the CF spectrum by combining prefrailty or frailty and pre-MCI (subjective cognitive decline) into reversible cognitive frailty (RCF), while the original CF as a combination of prefrailty or frailty and MCI is considered to be potentially RCF (PRCF) [8]. Nonetheless, information is limited on determinants of RCF or asymptomatic preclinical CF for identifying effective strategies for delaying the progression of onset of physical frailty and cognitive impairment.

While most studies have focused on associated factors with prevalent CF [9], very few emphasize on predictors of incident CF [10, 11], in which several modifiable risk factors, such as functional mobility, multimorbidity, fall history, low vitamin D, life satisfaction, depression, and processing speed, have been reported. On the other hand, gait characteristics, which may predict sarcopenia, falls, hospitalization, disability, and mortality [12, 13], are described as indicators of physical frailty and prodromal dementia [14]. A slow gait may predict cognitive decline and dementia [15], and gait variability is associated with cognitive function [16] but displays higher sensitivity than gait speed at predicting the occurrence of fall-related problems [17]. Nonetheless, to our knowledge, no study has investigated relationships of gait variability with CF.

We conducted a 2-year prospective study to investigate potential predictors, including gait characteristics, of incident RCF and PRCF in Taiwanese older adults.

## Methods

### Study participants

**Fig. 1:**
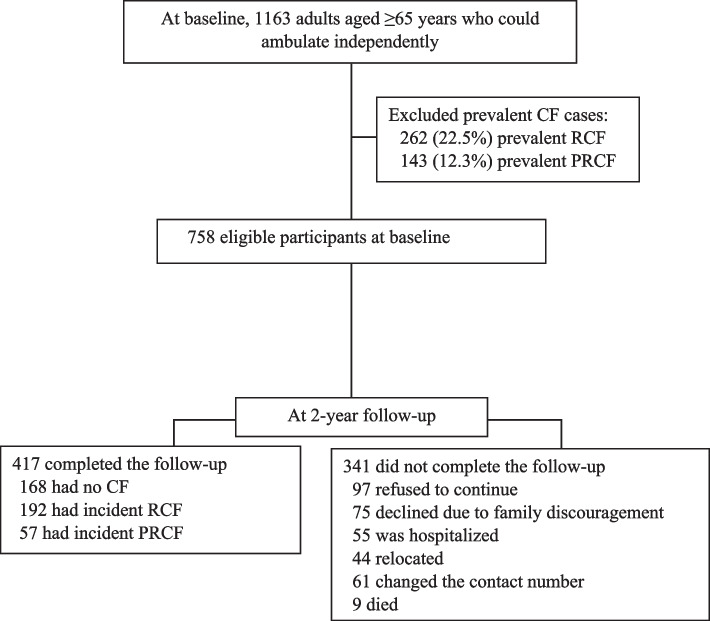
Flow diagram of study participants who developed three cognitive frailty levels of no cognitive frailty (CF), reversible CF (RCF), and potentially RCF (PRCF) during a 2-year study period

This study was a prospective cohort design. During the period of August 2017 to December 2018, we recruited participants from among those who visited outpatient clinics at Taipei Medical University Hospital in Taipei, Taiwan. Individuals who were aged ≥ 65 years, could ambulate independently, and did not have RCF or PRCF were eligible for the study. Conversely, individuals who could not independently perform basic activities of daily living (ADLs), had communication difficulties, or had major health problems (e.g., advanced cancer, major cardiopulmonary disease, or dementia) were excluded from the baseline assessment. Individuals who refused or were unable to undergo the follow-up assessment due to poor health, family issues, weather conditions, or transportation inconvenience, were also excluded from the study. The research protocol was approved by the Institutional Review Board of Taipei Medical University, and written informed consent was obtained from each participant. Figure 1 presents the flow diagram of study participants at the baseline and the 2-year follow-up assessment.

### Baseline assessment

The baseline assessment collected information on physical frailty and cognitive measures, as well as sociodemographic and lifestyle characteristics, preexisting comorbidities and medications, gait characteristics, balance measures, ADLs, and depressive symptoms.

### Frailty measures

Phenotype frailty comprised of five components of weight loss, exhaustion, low physical activity, slowness, and weakness was measured [18]. Unintentional weight loss was defined as > 3 kg or 5% of body weight over the past year. Muscle weakness was assessed by the grip strength of the right hand using a handgrip dynamometer and was measured in kilograms of isometric force. Low grip strength was gender-specifically defined to be ≤ 29 kg for males and ≤ 17 kg for females [18]. Self-reported exhaustion was determined as a positive response to a question (“I felt that everything I did was an effort”). Slowness was defined as a gait velocity of < 0.8 m/s at a normal walking pace. The International Physical Activity Questionnaire-Short Form (IPAQ-SF) was used to quantify physical activities in daily life [19]; low physical activity was defined as having fewer than 3 days of vigorous-intensity activity for at least 20 min/day or < 5 days of moderate-intensity activity or walking for at least 30 min/day. The presence of three or more of the five components was considered to indicate frailty, one or two components as pre-frailty, and the absence of all five components as non-frailty.

### Cognitive measures

The Mini-Mental State Examination (MMSE) evaluates orientation, registration, recall of information, attention and calculation, language, and visuospatial construction, with a score range of 0 to 30 [20, 21]. The MMSE was used to assess global cognitive function. The Clinical Dementia Rating (CDR) assesses cognitive performance in six domains: memory, orientation, judgment and problem solving, community affairs, home and hobbies, and personal care. The global CDR is a 5-point scale, with 0 indicating no impairment, 0.5 questionable, 1 mild, 2 moderate, and 3 severe cognitive impairment [22]. The global CDR was used to assess the cognitive status for determining RCF, PRCF, and dementia.

The Mattis Dementia Rating Scale (MDRS) has 36 items, designed to evaluate five cognitive domains: attention, initiation/perseveration, construction, conceptualization, and memory [23, 24]. Specifically, the five domains in sequence assess concentration, verbal fluency and initiation, visuospatial skills, analogical reasoning and associative thinking, and short-term memory, and score ranges for each domain are 0 ~ 37, 0 ~ 37, 0 ~ 6, 0 ~ 39, and 0 ~ 25 points. The Digit Symbol Substitution Test (DSST), consisting of a series of numbers and corresponding symbols, measures general and unspecific processing speed [25]. In the DSST, a participant was instructed to fill in a response form with as many corresponding symbols to numbers as possible in 90 s, and the number of correct number-symbol matches was recorded. The MDRS and DSST were used to assess six specific domains of cognition.

### Determination of CF

Frailty states and cognitive functions were followed up for 2 years after the baseline assessment to determine incident RCF and PRCF that occurred in the study period. Incident RCF was defined as the presence of cognitive impairment by subjective cognitive decline with pre-frailty/frailty, and incident PRCF was defined as the presence of MCI with pre-frailty/frailty [8]. The subjective cognitive decline was indicated when a participant had a CDR score equal to 0 and a positive response to an item (“Do you feel that you have more problems with thinking and memory than most?”), and MCI was based on the CDR being equal to 0.5. Otherwise, individuals who sustained normal cognition with non-frailty, pre-frailty or frailty only, subjective cognitive decline only, or MCI only were considered not to have CF (no-CF).

### Covariates

Sociodemographics and lifestyle behaviors consisted of age, gender, body-mass index (BMI), educational level, monthly household income, regular exercise habits, current smoking, and alcohol consumption. The BMI was calculated as the weight (kg) divided by the height squared (m^2^) and participants were categorized as being underweight (< 18.5 kg/m^2^), having a normal weight (18.5 ~ 22.9 kg/m^2^), being overweight (23 ~ 24.9 kg/m^2^), and being obese (≥ 25 kg/m^2^) [26]. Preexisting comorbidities were assessed using a list of 12 chronic conditions (hypertension, diabetes, heart disease, malignant tumors, respiratory tract disease, arthritis or rheumatism, gastric ulcers, liver disease, cataracts, kidney disease, gout, and spinal spurs), and medication use for any chronic conditions was documented.

Eight spatiotemporal gait characteristics, including velocity (cm/s), cadence (steps/min), step width (cm), stride length (cm), stride length variability (%), stride time variability (%), swing time variability (%), and double-support time variability (%), were assessed with a 6-m GAITRite electronic walkway (CIR Systems, Franklin, NJ, USA). Each participant was asked to walk on the GAITRite walkway at his/her usual pace [27], and the average of two trials was used for each gait characteristic. Before the formal trials, each participant practiced at least once. The coefficient of variation for variability variables was calculated.

Balance measures consisted of the Tinetti balance test and Activities-specific Balance Confidence (ABC) scale. The Tinetti balance test consists of 13 maneuvers used during daily activities and assesses static and dynamic balance ability; the balance score ranges 0 to 24, with a higher score indicating a greater balance ability [28]. The ABC scale assesses an individual’s confidence in performing 16 common daily tasks without losing their balance [29, 30]. The total score of the ABC scale is determined by the accumulated average of each item score and ranges 0 to 100, with lower scores indicating greater balance confidence or less fear of falling.

The Older Adults Resources and Services (OARS) ADL scale assesses seven basic ADLs and seven instrumental ADLs [31]. All items were graded as 2 (unable to perform the activity), 1 (needs some help), or 0 points (no help needed). The ADL score ranges 0 to 28, with a higher score indicating greater physical dependence. Depressive symptoms were assessed using the 15-item Geriatric Depression Scale (GDS), with a score of > 5 being indicative of depression [32].

### Statistical analysis

Baseline characteristics were compared among the three groups of RCF, PRCF, and no-CF, using analysis of variance (ANOVA) tests for continuous variables and Pearson’s Chi-squared tests for categorical variables. Baseline characteristics between participants who completed the follow-up and those who did not were compared to examine whether to have selection bias in the study.

We treated the three CF categories as nominal data, and a multinomial logistic regression model was performed to investigate independent associations of potential predictors with RCF and PRCF, using odds ratios (ORs) and 95% confidence intervals (CIs). To avoid large type-II errors in variable selection, variables with a *p* value of < 0.2 in the bivariable analyses were shortlisted into the initial multivariable analyses. In the multivariable analysis, stepwise selection was used, and variables with a *p* value of < 0.05 were retained in the final model. Age and sex were retained in the final model because of their clinical importance to physical frailty and cognitive decline. Moreover, a sensitivity analysis was conducted using a proportional odds model to check the results of the multinomial logistic model. The proportional odds model, which assumes that the effect of one exposure variable is the same across cumulative logits, treated the three CF categories as an ordinal scale. All data analyses were performed using SPSS Statistics vers. 25.0 for Windows (IBM, Armonk, NY, USA).

## Results

At the baseline, 758 older adults who did not have RCF or PRCF were eligible for this study. Over the 2-year study period, 417 eligible participants completed the follow-up assessment, among whom 192 developed incident RCF, 57 developed incident PRCF, and 168 remained without CF. Compared to the participants who completed the follow-up, those who did not complete it were significantly more likely to be older (mean age: 71.9 vs. 71.3 years), as well as to have a higher proportion of at least four comorbidities (29.0% vs. 17.0%), a higher proportion of at least four medications (39.6% vs. 25.4%), and depressive status (14.1% vs. 8.2%) at the baseline.

Table 1 presents the distributions of baseline characteristics among the three groups of no-CF, RCF, and PRCF. Among the three study groups, age, educational level, number of comorbidities, Tinetti balance, ABC scores, ADL scores, depressive status, MMSE scores, MDRS’s conceptualization and memory, DSST scores, and gait characteristics of velocity, cadence, step width, stride length, stride length variability, stride time variability, swing time variability, and double-support time variability significantly differed.

**Table 1:** Baseline characteristics of 168 participants who did not develop cognitive frailty (CF),192 who developed reversible CF (RCF), and 57 who developed potentially RCF (PRCF) over the 2-year study period

Characteristic	All *(N* = 417) mean ± SD or *n* (%)	No CF *(N* = 168) mean ± SD or *n* (%)	RCF *(N* = 192) mean ± SD or *n* (%)	PRCF *(N* = 57) mean ± SD or *n* (%)	*p* value
Age (years)	71.3 ± 5.5	70.1 ± 4.4	71.6 ± 5.8	74.1 ± 6.3	< 0.001
Sex					
Male	182 (43.6)	83 (49.4)	81 (42.2)	18 (31.6)	0.055
Female	235 (56.4)	85 (50.6)	111 (57.8)	39 (68.4)	
Body-mass index (kg/m^2^)					
Underweight (< 18.5)	23 (5.5)	7 (5.5)	14 (7.3)	2 (3.5)	0.835
Normal weight (18.5 ~ 22.9)	131 (31.4)	54 (32.1)	61 (31.8)	16 (28.1)	
Overweight (23 ~ 24.9)	118 (28.3)	47 (39.8)	53 (37.6)	18 (31.6)	
Obese (≥ 25)	145 (34.8)	60 (35.7)	64 (33.3)	21 (36.8)	
Educational level					
College or above	228 (54.7)	94 (56.0)	112 (58.3)	22 (38.6)	0.001
Senior and junior high	133 (31.9)	57 (33.9)	59 (30.7)	17 (29.8)	
Elementary or lower	56 (13.4)	17 (10.1)	21 (10.9)	18 (31.6)	
Income per month (NT$)					
Low (< 49.999)	194 (46.5)	69 (41.1)	91 (47.4)	34 (59.6)	0.067
Middle (50.000 ~ 99.999)	158 (37.9)	70 (41.7)	68 (35.4)	20 (35.1)	
High (≥ 100.000)	65 (15.6)	29 (17.3)	33 (17.2)	3 (5.3)	
Regular exercise (≥ 3 times per week)					
No	64 (15.3)	24 (14.3)	26 (13.5)	14 (24.6)	0.113
Yes	353 (84.7)	144 (85.7)	166 (86.5)	43 (75.4)	
Current smoking					
No	401 (96.2)	161 (95.8)	184 (95.8)	56 (98.2)	0.678
Yes	16 (3.8)	7 (4.2)	8 (4.2)	1 (1.8)	
Alcohol consumption					
No	358 (85.9)	141 (83.9)	164 (85.4)	53 (93.0)	0.231
Yes	59 (14.1)	27 (16.1)	28 (14.6)	4 (7.0)	
Number of comorbidities					
0 or 1	164 (39.3)	74 (44.0)	77 (40.1)	13 (22.8)	0.015
2 or 3	182 (43.6)	74 (44.0)	80 (41.7)	28 (49.1)	
≥ 4	71 (17.0)	20 (11.9)	35 (18.2)	16 (28.1)	
Number of medications					
0 or 1	99 (23.7)	40 (23.8)	47 (24.5)	12 (21.1)	0.145
2 or 3	212 (50.8)	95 (56.5)	92 (47.9)	25 (43.9)	
≥ 4	106 (25.4)	33 (19.6)	53 (27.6)	20 (35.1)	
Tinetti balance (0 ~ 24)	23.3 ± 1.4	23.4 ± 1.3	23.4 ± 1.0	22.6 ± 2.2	< 0.015
ABC scale (0 ~ 100)	86.9 ± 10.4	89.5 ± .8.1	86.2 ± 10.4	81.4 ± 13.6	< 0.001
ADL score					
28	404 (96.9)	166 (98.8)	187 (93.9)	51 (89.5)	0.002
< 28	13 (3.1)	2 (1.2)	5 (2.6)	6 (10.5)	
GDS score					
≤ 5	383 (91.8)	160 (95.2)	178 (92.7)	45 (78.9)	< 0.001
> 5	34 (8.2)	8 (4.8)	14 (7.3)	12 (21.1)	
Cognitive measures					
MMSE score	24.7 ± 2.4	25.3 ± 2.1	24.7 ± 2.1	23.1 ± 3.3	< 0.001
MDRS domains					
Attention	36.4 ± 0.9	36.5 ± 0.7	36.4 ± 0.9	36.2 ± 1.0	0.333
Conceptualization	36.4 ± 2.5	36.4 ± 2.6	36.6 ± 2.5	35.6 ± 2.4	0.006
Construction	5.7 ± 0.5	5.8 ± 0.5	5.7 ± 0.6	5.7 ± 0.6	0.382
Initiation/perseveration	33.3 ± 4.1	33.6 ± 3.9	33.3 ± 4.0	32.0 ± 4.9	0.081
Memory	23.5 ± 2.0	23.8 ± 1.9	23.5 ± 1.8	22.7 ± 2.8	0.019
DSST score	39.2 ± 12.8	41.2 ± 11.5	40.0 ± 12.7	30.2 ± 13.3	< 0.001
Gait characteristics					
Velocity (cm/s)	115.0 ± 21.8	120.5 ± 20.9	114.7 ± 20.2	100.0 ± 22.3	< 0.001
Cadence (steps/min)	111.1 ± 11.3	112.8 ± 10.8	111.0 ± 10.5	106.5 ± 13.8	0.001
Step width (cm)	63.2 ± 8.2	65.2 ± 7.8	63.1 ± 7.9	57.5 ± 8.2	< 0.001
Stride length (cm)	124.0 ± 16.9	128.3 ± 15.8	123.9 ± 16.2	112.2 ± 16.5	< 0.001
Stride length variability (%)	2.4 ± 2.0	2.2 ± 1.8	2.4 ± 2.1	3.0 ± 2.1	0.011
Stride time variability (%)	2.4 ± 1.8	2.2 ± 1.7	2.3 ± 1.9	3.3 ± 1.9	< 0.001
Swing time variability (%)	4.5 ± 3.5	4.4 ± 3.4	4.2 ± 3.3	6.1 ± 4.2	0.002
Double-support time variability (%)	5.7 ± 4.1	5.2 ± 3.6	5.9 ± 4.1	6.9 ± 5.1	0.041

*ABC* Activities-specific Balance Confidence, *ADL* Activity of daily living, *CDR* Clinical Dementia Rating, *CF* Cognitive frailty, *DSST* Digit symbol substitution test, *GDS* Geriatric Depression Scale, *MMSE* Mini-Mental Status Examination, *MDRS* Mattis Dementia Rating Scale, *NT$* New Taiwan dollar (in 2021, the average exchange rate was US1 ≈ *TW *30), *SD* Standard deviation

Table 2 presents results of the bivariable multinomial logistic analysis for incident RCF and PRCF over the study course. The development of RCF tended to be positively associated with an older age, lower scores on the ABC and MMSE, a slower gait, a smaller step width, and a smaller stride length. The development of PRCF tended to be positively associated with an older age, female sex, lower educational levels, lower BMI levels, a higher number of comorbidities, lower scores on the Tinetti balance, ABC, ADL, and GDS, lower scores on the MMSE, MDRS’s conceptualization, initiation/preservation, and memory, and DSST, a slower gait velocity, a lower cadence, a narrower step width, a shorter stride length, and greater variabilities of stride length, stride time, swing time, and double-support time.

**Table 2:** Results of bivariable multinomial logit model analyses of explanatory variables with odds ratios (ORs) and 95% confidence intervals (CIs) for predicting incident reversible cognitive frailty (RCF) and potentially RCF (PRCF) over the 2-year study period

Characteristic	RCF OR (95% CI)	*p* value	PRCF OR (95% CI)	*p* value
Age (years)	1.06 (1.02 ~ 1.11)	0.007	1.14 (1.08 ~ 1.20)	< 0.001
Women (vs. men)	1.34 (0.88 ~ 2.03)	0.171	2.12 (1.12 ~ 3.99)	0.021
Body-mass index				
Normal weight	1.00 (reference)		1.00 (reference)	
Underweight	1.77 (0.67 ~ 4.71)	0.252	0.96 (0.18 ~ 5.11)	0.966
Overweight and obese	0.97 (0.62 ~ 1.52)	0.887	1.23 (0.63 ~ 2.40)	0.543
Educational level				
College or above	1.00 (reference)		1.00 (reference)	
Senior and junior high	0.87 (0.55 ~ 1.37)	0.545	1.27 (0.62 ~ 2.60)	0.505
Elementary or lower	1.04 (0.52 ~ 2.08)	0.919	4.52 (2.01 ~ 10.2)	< 0.001
Income per month				
Low	1.00 (reference)		1.00 (reference)	
Middle	0.74 (0.47 ~ 1.16)	0.190	0.58 (0.30 ~ 1.11)	0.098
High	0.86 (0.48 ~ 1.56)	0.623	0.21 (0.06 ~ 0.74)	0.015
Regular exercise (≥ 3 times per week)	1.06 (0.59 ~ 1.94)	0.839	0.51 (0.24 ~ 1.08)	0.077
Current smoking (vs. no)	1.00 (0.36 ~ 2.82)	1.000	0.41 (0.05 ~ 3.41)	0.410
Alcohol consumption (vs. no)	0.89 (0.50 ~ 1.58)	0.696	0.39 (0.13 ~ 1.18)	0.096
Number of comorbidities				
0 or 1	1.00 (reference)		1.00 (reference)	
2 or 3	1.04 (0.66 ~ 1.63)	0.868	2.15 (1.04 ~ 4.48)	0.040
≥ 4	1.68 (0.89 ~ 3.18)	0.109	4.55 (1.88 ~ 11.0)	0.001
Number of medications				
0 or 1	1.00 (reference)		1.00 (reference)	
2 or 3	0.82 (0.50 ~ 1.37)	0.457	0.88 (0.40 ~ 1.92)	0.742
≥ 4	1.37 (0.75 ~ 2.50)	0.312	2.02 (0.86 ~ 4.73)	0.105
Tinetti balance (0 ~ 24)	0.99 (0.82 ~ 1.19)	0.889	0.73 (0.60 ~ 0.89)	0.002
ABC scale (0 ~ 100)	0.96 (0.94 ~ 0.98)	0.001	0.93 (0.90 ~ 0.96)	< 0.001
ADL score (< 28 vs. ≥ 28)	2.22 (0.43 ~ 11.6)	0.345	9.77 (1.91 ~ 49.8)	0.006
GDS score (> 5 vs. ≤ 5)	1.57 (0.64 ~ 3.85)	0.321	5.33 (2.06 ~ 13.8)	0.001
Cognitive measure				
MMSE score	0.88 (0.79 ~ 0.97)	0.014	0.69 (0.61 ~ 0.79)	< 0.001
MDRS domains				
Attention	0.87 (0.67 ~ 1.11)	0.259	0.74 (0.53 ~ 1.03)	0.071
Conceptualization	1.02 (0.94 ~ 1.11)	0.627	0.89 (0.80 ~ 0.99)	0.039
Construction	0.88 (0.59 ~ 1.30)	0.512	0.75 (0.45 ~ 1.27)	0.284
Initiation/perseveration	0.98 (0.93 ~ 1.03)	0.385	0.92 (0.86 ~ 0.98)	0.012
Memory	0.92 (0.82 ~ 1.03)	0.144	0.79 (0.89 ~ 0.91)	0.001
DSST score	0.99 (0.98 ~ 1.01)	0.336	0.93 (0.91 ~ 0.96)	< 0.001
Gait characteristics				
Velocity (cm/s)	0.99 (0.98 ~ 0.99)	0.009	0.95 (0.94 ~ 0.97)	< 0.001
Cadence (steps/min)	0.99 (0.97 ~ 1.00)	0.112	0.95 (0.93 ~ 0.98)	< 0.001
Step width (cm)	0.97 (0.94 ~ 0.99)	0.012	0.88 (0.84 ~ 0.92)	< 0.001
Stride length (cm)	0.98 (0.97 ~ 0.99)	0.011	0.94 (0.92 ~ 0.96)	< 0.001
Stride length variability (%)	1.06 (0.95 ~ 1.18)	0.336	1.19 (1.04 ~ 1.37)	0.013
Stride time variability (%)	1.01 (0.89 ~ 1.15)	0.849	1.30 (1.11 ~ 1.51)	0.001
Swing time variability (%)	0.99 (0.93 ~ 1.05)	0.692	1.12 (1.04 ~ 1.21)	0.003
Double-support time variability (%)	1.05 (0.99 ~ 1.11)	0.070	1.11 (1.03 ~ 1.19)	0.006

*ABC* Activities-specific Balance Confidence, *ADL* Activity of daily living, *GDS* Geriatric Depression Scale, *MMSE* Mini-Mental Status Examination, *MDRS* Mattis Dementia Rating Scale

Results of the multinomial logistic regression analysis for incident RCF and PRCF over the study are shown in Table 3. After adjusting for sex, incident RCF was significantly associated with an older age (OR = 1.05 and 95% CI = 1.00 ~ 1.10) and lower ABC scores (OR = 0.97 and 95% CI = 0.95 ~ 0.99). Furthermore, incident PRCF was significantly associated with an older age (OR = 1.07 and 95% CI = 1.01 ~ 1.14), lower ABC scores (OR = 0.96 and 95% CI = 0.93 ~ 0.99), the presence of depression (OR = 3.61 and 95% CI = 1.21 ~ 10.8), lower MMSE scores (OR = 0.83 and 95% CI = 0.72 ~ 0.96), a slower gait velocity (OR = 0.97 and 95% CI = 0.96 ~ 0.99), and greater double-support time variability (OR = 1.09 and 95% CI = 1.01 ~ 1.18).

**Table 3:** Result of the multivariable multinomial logit model analyses of explanatory variables with odds ratios (ORs) and 95% confidence intervals (CIs) for predicting incident reversible cognitive frailty (RCF) and potentially RCF (PRCF) over the 2-year study period

Characteristic	RCF OR (95% CI)	*p* value	PRCF OR (95% CI)	*p* value
Age (years)	1.05 (1.00 ~ 1.10)	0.049	1.07 (1.01 ~ 1.14)	0.039
Women (vs. men)	1.33 (0.86 ~ 2.07)	0.199	1.86 (0.90 ~ 3.81)	0.092
ABC scale (0 ~ 100)	0.97 (0.95 ~ 0.99)	0.015	0.96 (0.93 ~ 0.99)	0.010
GDS score (> 5 vs. ≤ 5)	1.39 (0.55 ~ 3.55)	0.487	3.61 (1.21 ~ 10.8)	0.021
Cognitive measure				
MMSE score	0.95 (0.85 ~ 1.06)	0.323	0.83 (0.72 ~ 0.96)	0.015
Gait characteristics				
Gait velocity (cm/s)	0.99 (0.98 ~ 1.00)	0.223	0.97 (0.96 ~ 0.99)	0.002
Double-support time variability (%)	1.05 (0.99 ~ 1.11)	0.082	1.09 (1.01 ~ 1.18)	0.038

*ABC* Activities-specific Balance Confidence, *GDS* Geriatric Depression Scale, *MMSE* Mini-Mental Status Examination

The sensitivity analysis from the proportional odds model showed similar results with the exception that the association of female sex and higher levels of CF was significant. These results indicated that an older age (OR = 1.05 and 95% CI = 1.01 ~ 1.09), female sex (OR = 1.50 and 95% CI = 1.01 ~ 2.23), lower scores on the ABC (OR = 0.97 and 95% CI = 0.95 ~ 0.99) and MMSE (OR = 0.89 and 95% CI = 0.81 ~ 0.97), the presence of depression (OR = 2.34 and 95% CI = 1.15 ~ 4.73), a slower gait velocity (OR = 0.99 and 95% CI = 0.98 ~ 0.99), and greater double-support time variability (OR = 1.06 and 95% CI = 1.01 ~ 1.11) were significantly associated with a higher risk of developing RCF or PRCF over the study period.

## Discussion

In summary, incident RCF was significantly associated with an older age and lower balance confidence, and incident PRCF was significantly associated with an older age, female sex, lower balance confidence, reduced global cognition, the presence of depressive symptoms, a slower gait velocity, and greater double-support time variability.

As with previous studies [33, 34], an older age was associated with higher risks of RCF and PRCF. There is a higher risk of developing dementia and mortality for older adults with CF vs. those with physical frailty or cognitive impairment alone [35], indicating that the presence of CF could add additional risk of adverse health outcomes, and CF might not be a normal part of the aging process. On the other hand, a recent study based on electroencephalograms, magnetoencephalograms, and magnetic resonance imaging (MRI) supported that CF should represent a spectrum of normal cognitive aging rather than incipient or undiagnosed Alzheimer's disease (AD) [36], in which cognitively frail older adults and cognitively intact older adults showed a stronger physiological mismatch (induced by a cross-modal paired-associates task that presented images followed by sounds) and larger temporal gray matter volumes than those with MCI and AD. It is possible that abnormal and normal processes of cognitive aging can coexist.

In this study, low balance confidence or fear of falling was the only modifiable factor associated with the development of both RCF and PRCF, implying that balance confidence might be a sensitive indicator for detecting early-stage CF and predicting late-stage CF. Fear of falling is common among older adults, and a severe fear of falling might not only be associated with a reduction in physical activity and mobility, and increase physical frailty, gait dysfunction, and fall-related problems [37, 38], but also have an impact on cognitive decline [39]. The mechanisms of fear of falling in terms of cognitive function are probably through physical inactivity [40], depressive symptoms [34], and lower social participation [41]. Since this result was controlled for depression status, global cognition, and gait characteristics, the effect of balance confidence on PRCF might not result from physical inactivity and low social participation, although further studies are needed. On the other hand, fear of falling might not just be an acute outcome resulting from a fall; once fear develops, it is likely to persist, regardless of whether there is a fall. For instance, a study reported that individuals who expressed fear of falling at the baseline were more than 5-times as likely to express fear at the 20-month follow-up [42]. Therefore, as an early intervention for CF, enhancing balance confidence might be a target to delay the onset and progression of physical frailty and cognitive impairment. Among various interventions of CF, multidomain interventions, which often consist of multicomponent exercise, cognitive training, dietary counseling, and psychosocial supports [43, 44], might be promising to increase balance confidence and eventually reverse RCF and PRCF in older people.

Depression was associated with incident PRCF but not with incident RCF in our study, indicating that depressive symptoms might not be sensitive enough to detect early-stage CF. The relationship of depression with CF can be influenced or confounded by certain factors such as the use of antidepressants [45], cerebrovascular disease, and other factors [46]. However, our result about depression remained significant after antidepressant use was adjusted for (data not shown). It was found that depression may be independently associated with deterioration of one's physical ability which may further cause changes in physical activity, cognitive functioning, and social involvement, thereby leading to rapid progression into adverse conditions in older adults [47]. A large longitudinal study of older men even exhibited a temporal dose–response relationship between depressive symptoms and dementia [48]. Therefore, depression could be a risk factor for physical frailty and cognitive impairment but might not be predictive of prefrailty and subjective cognitive decline.

Lower global cognition at the baseline was associated with incident PRCF but not incident RCF. Lower global cognition was associated with functional disabilities [49] and a slower gait speed [50], but cognitive decline is seldom reported as a risk factor for both physical and cognitive frailty [5]. On the other hand, physical frailty was found to increase the risks of MCI, cognitive decline, and all-cause dementia in cognitively intact people [51], and even its three components of exhaustion, slowness, and inactivity were independently associated with lower global cognition [52]. Those findings imply that physical frailty might have stronger impacts on the early development of CF than does cognitive decline, although incident dementia might occur more frequently in those who develop cognitive impairment before frailty than those who develop frailty before cognitive impairment [53]. Moreover, after adjusting for global cognition, the associations between certain cognitive domains and incident PRCF become non-significant, indicating the effect of aging on multiple cognitive domains and the importance of cognitive reserve for preventing or delaying frailty and cognitive impairment.

Gait velocity and double-support time variability were significantly associated with incident PRCF but not RCF. Here, the insensitivity of gait characteristics in terms of RCF is probably because prevalent RCF cases had been excluded from study eligibility and asymptomatic RCF cases were still in the very early preclinical stage. A slower gait is consistently associated with cognitive declines and dementia [12, 13, 15] and specifically parallels declines in global cognition, memory, and executive function [54]. Furthermore, as one of the early motor dysfunctions, a slow gait may discriminate between non-frail and pre-frail/frail states [12, 14] and may coexist with or precede the onset of cognitive decline and correlated brain amyloidosis [55]. To maintain physical and cognitive abilities, older adults are recommended to walk at a speed of ≥ 100 steps/min for moderate-intensity physical activity (i.e., brisk walking) [56]; nevertheless, whether intensive physical activity can reverse PRCF becoming RCF or even no-CF remains to be explored. Increased double-support time variability, which reflects a reduction in the lateral postural stability and single-limb balance control, was also predictive of falls and impaired mobility [57], as well as cognitive decline and dementia [54, 58], particularly with greater declines in memory function [59]. Although gait variability seems to be a more-sensitive predictor for the occurrence of a fall than gait speed [17], larger double-support time variability could also result from a strategic use of double support to re-stabilize after balance perturbations [60]. Therefore, it is necessary to investigate differences in changes in double-support time variability among different challenges to mobility and across different levels of CF.

There are several limitations to our study. First, further investigation is needed to examine whether the associations between the predictors and incident RCF and PRCF were causal, since our follow-up time was short and some unobserved variables, particularly psychosocial factors, might have confounded the results. Second, a substantial proportion of subjects did not complete the follow-up, and they were more likely to be older and have a higher number of comorbidities and depression, which are related to the development of physical frailty and cognitive impairment. Hence, a healthy survival effect might have existed in those who completed the follow-up that could have led to reduced numbers of RCF and PRCF cases, thereby underestimating the effects of these variables. Third, generalizing our findings to those older adults who are healthier and with lower risk of chronic diseases should be done mindfully because our participants were recruited from outpatient clinics of a general hospital that might not be fully representative of community-dwelling older people. Finally, our straight level-ground walkway for participants was only 6 m long, and increasing the walking distance could possibly provide more-reliable estimates of the gait variability measures.

## Conclusions

This study demonstrated that an older age, lower balance confidence, reduced global cognition, the presence of depressive symptoms, a slower gait velocity, and greater double-support time variability may increase the risk of incident PRCF, while only an older age and lower balance confidence were independently associated with incident RCF. Balance confidence has the potential to be an indicator for detecting early-stage CF and predicting late-stage CF.

## Data Availability

The datasets generated and analyzed during the current study are not publicly available due to ethical restrictions and patient confidentiality but are available from the corresponding author upon reasonable request.

## Abbreviations

ABC: Activities-specific Balance Confidence
AD: Alzheimer's disease
ADL: Activity of daily living
BMI: Body-mass index
CI: Confidence interval
CDR: Clinical Dementia Rating
CF: Cognitive frailty
DSST: Digit Symbol Substitution Test
MCI: Mild cognitive impairment
MMSE: Mini-mental state examination
PRCF: Potentially reversible cognitive frailty
RCF: Reversible cognitive frailty
SD: Standard deviation
